# On the Morbid Effects of an Oppressed Brain

**Published:** 1819-09

**Authors:** 


THE LONDON

### Medical and Physical Journal.

3 OF VOL. XLII.] SEPTEMBER, 1819. [NO. 247.

"For many fortunate discoveries in medicine, and for the detection of numerous "errors, the world is indebted to the rapid circulation of Monthly Journals; "and there never existed any work to which the Faculty in EUROPE and "AMERICA were under deeper obligations than to the Medical and Physical "Journal of London, now forming a long, but an invaluable, series." RUSH.

### Original Communications.

FOR THE LONDON MEDICAL AND PHYSICAL JOURNAL.

### *On the Morbid Effects of an Oppressed Brain.* BY DR. KINGLAKE.

THE healthful functions of the brain are so extensive and complicated, that not the slightest deviation can happen from the natural state of that organ, without inducing a greater or less degree of disease. Constituting as it does in the animal economy the grand source of vital energy, it cannot in any measure be disordered, without being attended by morbid effects characteristic of the particular ailment that may have occurred. The provisions of nature are large for its security; its mechanical defences are of the most appropriate and efficient kind: but, with all this natural immunity, the brain is liable to great, manifold, and irremediable injuries, from various causes. It is not intended, at present, to discuss the mischiefs that may arise from external violence, but more particularly to advert to some of the leading morbid effects that result from the influence of internal causes.

An oppressed state of brain is at all times a disease of vast moment. However it may have been induced, it is enough to know that it actually exists, to require the earliest and most unsparing endeavours for its timely removal. In this inquiry, it is material to distinguish between the instances of oppressed brain from causes originating in the cerebral organ itself, and such as may have sympathetically extended the morbid effect from other parts of the system. The effects resulting from these primary and secondary causes of disease may not be widely different, yet it would be practically important to discriminate the one from the other, for the purpose of more immediately applying the proper remedy to the affected part. An oppressed brain may directly originate from either increased arterial action, congestive fullness of vessel, extravasated fluids, or from diminished nervous energy. In the first place, the augmented impetus of blood to the brain would be apt to induce severe head-ach, watchfulness, and raving delirium; whilst, in the three latter, stupor and comparative insensibility would probably arise. In the former case, the system at large sympathizing with the affected brain, would assume an excessive degree of arterial action; but, in the latter instances, torpor and deficient excitement, would restrain and depress the active energies of life.

These two opposite states equally derange the nervous system, and tend to induce deep, extensive, and various disease, throughout the whole frame. Without a due degree of nervous energy, it is not possible for either the bodily or mental functions to be healthfully performed. Disorder and confusion are in the train of the irregular action occasioned by either excessive or deficient excitement of nervous influence. Lodged in a bony cavity, and supplied with blood through arteries directed in a tortuous course, the brain is secured from injury from slight causes. Circumstances must be unfavourable, and must concur in producing considerable violence, before an hurtful impression can be made on the great citadel of life and health. When, however, an inroad has been made on its healthful condition, when the distinct and connected harmonies of its power and efficiency are disconcerted, the consequences are afflictingly experienced, in diseases corresponding to the particular nature and influence of the offensive agency that may have been exerted. When an oppressed state of the brain has so stimulated the sensorial power as to have induced agonizing pains in the head, and to have awakened painful sympathies in other parts of the body, the disease must be regarded as one of immoderate nervous excitement, in which the sensibility, which in natural circumstances would be undisturbed, is here distempered and thrown into agitations and commotions, that constitute acute affections of the nervous system of the worst form. The ferocious kinds of mania and of phrenzy are examples of this severe affection.

In these cases, the sensorial power is not only inordinately excited, but is also so vitiated as to induce painful feelings, that are never experienced but as the effects of morbid sensibility. The vast range which the sphere of feeling affords, is closely occupied by the endless variety of sensation which the disordered state of nervous influence is capable of generating; feelings, indeed, which have no existence in the natural state of things, and which grow out of the distempered condition in which the organs of sense are placed. The excessive action which lies at the root of this disordered feeling should be subdued without delay, lest it should establish itself by habit, and ultimately render incurable, what, at an earlier period, might have been radically overcome.

Persons of robust stamina, whose constitutions are unbroken, and who have been exposed to the pernicious influence of strong and distressful impressions, whose passions of mind have been intemperately acted on, who have been subjected to situations of keen and afflicting trial, and who have had to sustain protracted conflicts in unequal and disadvantageous contests, are those who are apt to become the unhappy victims of morbid sensibility connected with immoderate excitement. The brain, in such circumstances, is oppressed, but not so as to depress its vital energies: on the contrary, they are greatly excited by the vascular fulness that prevails. In the instances in which congestive plenitude has obtained in the brain, by which the active energies of the nervous system are depressed, the injury that ensues is less formidable in apparent violence, but equally dangerous in its probable issue. Whether nervous energy be excessively or deficiently exerted, the morbid effects of either state are strikingly manifested. Active or passive mischief is the direct result: in the latter case, torpor becomes the unwieldy and unimpressible obstacle to relief. Where a remedy can make no impression, no effect can be produced. This is the case in both the local and general torpor occurring in the brain and nervous system, when the cerebral organ is oppressed by a too tardy motion and congestive accumulation of the circulating fluids in its substance. This oppressed state, with insufficient energy to re-act and propel the lingering fluids, forms a prolific source of nervous diseases. They present, under an almost infinite variety of aspects and shades, from a sense of languor and dejection of mind, to corroding melancholy, despondent mania, apoplexy, palsy, and syncope. The gradations between these several states of disease are numerous, and may be justly said to comprehend the vast trains of nervous diseases that have equally embarrassed the nosologist to class, and the patient to describe.

If an oppressed brain from diminished energy be allowed to remain, its morbid effects will not be confined to the more immediate derangement of the nervous system, but will, through ganglion, and other sympathetic influence, disorder the functions of other viscera. The heart will partake in the mischief, becoming, according to circumstances, either unduly irritable or morbidly torpid. Its action will be either too quick and irregular, or too slow and irregular: in either case, a palpitating labour at the organ itself, and a disordered arterial circulation, will be the consequence.

The stomach and digestive organs will also soon share in the torpifying effects of an oppressed brain: the gastric and enteric juices will be deteriorated, and the digestive, assimilative, and chylipetive processes will be rendered imperfect, and unequal to supplying the system with such nourishment as the exigencies of life and health may require. The liver, lungs, kidneys, urinary bladder, &c. will soon participate in the paralyzing influence of an oppressed nervous energy. These viscera will be severally disabled in their respective functions. The biliary secretion, respiration, the formation of urine, its expulsion and retention, will be distinctly or collectively affected, as the weight of torpor and inaction may more particularly fall on one or all of these organs.

Although the brain may be justly regarded as the source of nervous power, as the main organ of sensation, from which the mandates of the will are issued and transmitted to all and every part of the frame; and to which the sensations of the same whole are referred, and by which they are recognized; yet it must be allowed, that the spinal marrow, the various pairs of nerves, and more especially their ganglion or confluent points, are also sentient portions, from which emanate no small share of nervous influence. It is to those parts of the nervous system that must be attributed the morbid effects often insidiously produced on the brain, by a deranged state of the first passages. It is well known that disordered digestion will so affect the sentient nerves of the stomach, as to induce vertigo, and even apoplectic abolition of all power of sense and motion. The heart also will intermit in its action, pending dyspeptic oppression of stomach.

An undue detention of venous blood in the liver, owing to a deficient secretion of bile; obstructed respiration, whether from glandular enlargement, vascular torpor, or serous effusion; renal inaction, surcharging the circulating fluids with the superfluous water destined to be removed by urinary secretion; or diminished perspiration; may so overburthen the sanguiferous vessels, as to occasion serious, and even destructive, oppression on the brain. It is by no means an unfrequent occurrence, for patients who are supposed, from impeded respiration, to have water in the cavity of the thorax, whether in the cellular structure of the lungs or more at large in the pleuretic bag, to be attacked suddenly with a fit of apoplexy. The venous plethora that results from the obstacle that is opposed to the blood in its passage through the lungs, must very directly have the effect of loading the brain to an extent that will sufficiently explain the destructive attack of apoplexy that occasionally arises from it.

In all cases of disease from an oppressed brain, founded in torpor or diminished vital energy, it is obvious that the endeavour should be to remove the hurtful cause in the most direct manner. In these instances, the morbid state is not so much owing to exhaustion of power as to its depression: it would therefore be injurious to resort to the use of stimulants, in the calculation of salutarily exciting the torpid state under which the patient appears to suffer. That mode of treatment would be likely to aggravate the mischief, by exciting vascular action, and propelling an additional incumbrance on the oppressed brain. The indication of cure, then, is so far to deplenish the vessels, by bleeding and procuring evacuations from the bowels, as to relieve the load already accumulated, and to enable the torpid vessels to exert such transmitting action as may prevent a recurrence of the oppressive fulness. With the removal of the oppressing weight from the brain the nervous energy will be restored, and renewed strength and harmony will be distributed and re-established throughout the system.

Abstinence from fermented liquors and from full meals will be essentially requisite, either in obviating a primary accession, or in preventing the recurrence of the morbid effects of an oppressed brain. In all visceral diseases connected with any degree of obstruction to the circulating fluids, vascular depletion, both by blood-letting and sufficient alvine discharges, should be esteemed indispensable. If these means be not timely employed, much danger will exist of the patient's being cut off, either by an apoplectic attack, by spasmodic difficulty of breathing, by suffocating hemoptöe, or by syncope. The indiscriminate adoption of tonic and stimulating treatment, in what are regarded as nervous affections, and in chronic visceral diseases, has had its hecatombs of victims. Instead of relieving vascular fulness, and other causes depressing and rendering torpid the active energies of life, under the delusive bugbear *debility*, a well-intended, but often destructive, attempt has been made to renovate the lost strength, by the very means that must have powerfully aided in additionally oppressing, and finally in extinguishing, life itself. Indeed, whatever distinctions may be made as to the morbid modes and conditions by which apoplexy may be induced, the effect in every instance is so much the same as to require, for the most part, similar treatment. The oppressed state of the brain, in every example of this disease, requires the most prompt and efficient relief. Delay is not only dangerous, but almost necessarily destructive. Nervous energy may be depressed, and even considerably exhausted, compatibly with life; but cannot be durably prostrated by apoplectic pressure, without incurring certain death. Under the most favourable circumstances, the chance of rallying is too distant to rest any reasonable expectation on it: the mortality of this disease is in an appalling proportion to the recoveries which happen. My own experience has been considerable in this malady; but the cases, in my observation, that have terminated favourably are almost eclipsed by those which have ended

4

fatally. Practical opportunities for seeing much of apoplectic disease have convinced me that the most efficient mode of relieving it, is by ample bleeding. Drawing blood from the arterial system on this occasion would seem to be preferable to venous bleeding; as the former would more directly diminish the impetus of the circulation on the brain, than could be effected by the latter. The temporal artery is convenient for this purpose, and will generally be found to yield the utmost quantity of blood that it may be expedient to abstract. If difficulties should occur in obtaining blood from the temporal artery, it may be sufficiently taken from either the jugular or brachial veins. In three cases of apoplexy, treated by very copious bleeding at intervals of about eight hours, for several successive days, until consciousness, the power of speech, and voluntary motion, were restored, final recovery was effected. The bowels were also at the same time briskly excited and evacuated. Extensive blistering of the scalp was likewise employed. But in other instances, in which more moderate bleeding was practised, together with brisk cathartics and vesication on the head, no perceptible benefit was obtained, and the issue was mortal. It appears therefore fair to infer, that the main remedy in apoplectic cases, is early and extensive vascular depletion. The state of arterial action will afford a sure guide as to how far the practice may be carried. It will be found that the action of the heart and arteries will acquire increased freedom and force, as the repeated bleedings have the effect of lessening the stupefactive pressure existing on the brain. Generally speaking, it would perhaps be a safe practical rule to persist in bleeding to the extent of twelve ounces every six hours, whilst the state of the pulse will admit of it, until some beneficial effect be produced. The slightest appearance of returning consciousness, vision, power of speech, or of voluntary motion, should be a warranty to proceed in the use of the remedy as long as arterial action will bear it, and the recovering state of the disease may require its continued aid.

Vascular fulness, distension, and pressure, appear conjointly to produce the abolition of sense and power of motion occurring in apoplectic paroxysms. This state will paralyze the nervous system beyond retrieval, if it be suffered to continue longer than may be necessary to give effect to the remedies that may be employed. To make room in the sanguiferous vessels, and to cause them to contract on their diminished contents, by large and persevering bleedings, would seem to be the most reasonable mode of endeavouring to render effectual benefit. If arterial action should flag under the use of large and repeated bleedings to such a degree as to contra-indicate a farther prosecution of the treatment, the more topical mode of relieving on the same principle, by cupping between the shoulders and on the shaved scalp, would be advisable. It may be presumed, that unnecessary apprehension is entertained respecting extreme bleeding in apoplectic seizures. The usual quantity of blood that is taken away on these occasions would seem to be far short of that which may be borne without at all endangering the extinction of life, and much too small to induce the salutary depletion of the vascular system which the proposed remedy is intended to accomplish.

In addition to the three instances of the successful result of extensive bleedings in apoplectic attacks already cited, it may not be inapplicable to adduce a somewhat analogous case, that some time since occurred in my practice. A person of a sanguineous temperament, and accustomed to rather an intemperate mode of living, was suddenly seized with an oppression of the brain, by which consciousness and power of voluntary motion were nearly suspended. Bleeding had been repeatedly undergone previously to my being consulted, which, as well as my recollection serves me, was on the second day of the disease. In my judgment, farther bleeding was required; and, at my suggestion, the temporal artery was opened for that purpose. The blood flowed freely, and was drawn to the extent of about eighteen ounces. No discoverable relief however was derived, either from this or the former bleedings, until, in the course of the ensuing night after the arterial bleeding, the incised temporal artery spontaneously bled afresh, and so copiously, as probably to have induced a state of arterial inaction that restrained farther effusion. The quantity of blood lost on this occasion, judging from the appearance of the patient's body and bedlinen, must have been very large. It was however speedily followed by a return of natural consciousness, reason, speech, and power of voluntary motion, which soon terminated in complete recovery. The medical practitioner, who closely attended this case, and witnessed the occurrence stated, had no hesitation in ascribing the recovery to the very large quantity of blood which had thus accidentally escaped.

In farther support of the opinion that much larger, or rather more persevering, bleedings are necessary in affections of the brain than are commonly practised, a very late instance may be stated, of an injured brain from external violence, in which at first but little inconvenience was felt. After the lapse of a few weeks, however, symptoms of an oppressed brain were shown, by head-ach, giddiness, confused vision, and general prostration of strength. The pulse was feeble, small, and sluggish. Much inability for bodily exertion, and failure of mental energy, were experienced, altogether clearly denoting that the healthy function of the brain was considerably disordered. Bleeding and other evacuations had been carried to some extent, but had been perhaps too cautiously limited by the feelings of the patient, which were those of extreme weakness. Conceiving that this sense of debility arose from a depressed, and not from an exhausted, state of vital power, and that the offending cause was vascular fulness of the brain, no difficulty occurred to me in directing an ample bleeding from the temporal artery. This was done with sensible relief. The pulse became more free, active, and full, after the bleeding than before; yet violent head-ach continued: it would indeed remit, and sometimes even intermit; but this abatement was soon succeeded by an exasperated recurrence of pain. In one of the painful fits, the arterial impulse on the brain was so strong as to cause a renewed bleeding from the part of the temporal artery that had been opened, from which escaped a considerable quantity of blood, and with manifest benefit. A similar accident afterwards occurred in numerous instances, and with such advantageous consequences, that the patient regarded the unclosed orifice in the artery as a salutary fontanel; and, but for the precariousness of suffering blood to flow from such an awful source of supply, would have been desirous of keeping it open. It was not however until after many unavailing endeavours, that the artery could be either closed, or so compressed, as to prevent renewed effusions of blood. These were evidently occasioned by the increased vascular action attending the head-ach; nor did the orifice heal, nor were the spontaneous bleedings restrained, until the arterial action had resumed the healthy state, and the head-ach had almost wholly subsided. It is difficult to say how much blood escaped by those casual bleedings; it could only be estimated by the discoloured linen, by the frequency of the recurrence, and the abatement of pain resulting from them. These effusions altogether formed a series of bleeding that would hardly be risked in practice, and most certainly would not have been repeated so often, and to the extent that happened on these occasions, and by which an ultimate cure was effected. The patient progressively amended, is now well; and it may be fairly presumed, that the restoration of health is chiefly attributable to the large and seasonable flow of blood which was accidentally yielded by the unclosed opening in the temporal artery.

These two instances of renewed bleeding from the temporal artery having afforded such decided and effectual relief, are strong facts, and deserve, in conjunction with other reasonable authorities for free bleeding in apoplectic and other cases of oppressed brain, to have due weight in determining not only the general expediency of the practice on such occasions, but also how far it ought to be carried, to insure the salutary efficiency that it may be capable of producing.

*Taunton; June 12th,* 1819.

